# Milk Fatty Acids as Potential Biomarkers of Enteric Methane Emissions in Dairy Cattle: A Review

**DOI:** 10.3390/ani15152212

**Published:** 2025-07-28

**Authors:** Emily C. Youngmark, Jana Kraft

**Affiliations:** 1 Department of Animal and Veterinary Sciences, The University of Vermont, Burlington, VT 05405, USA; emily.youngmark@uvm.edu; 2 Department of Medicine, Division of Endocrinology, Metabolism and Diabetes, The University of Vermont, Colchester, VT 05446, USA; 3 Department of Nutrition and Food Sciences, The University of Vermont, Burlington, VT 05405, USA

**Keywords:** milk fatty acids, methanogenesis, climate change, greenhouse gases, methane measurement, milk biomarkers

## Abstract

Measuring methane (CH_4_) emissions from dairy systems is crucial for advancing sustainable agricultural practices aimed at mitigating climate change. However, current CH_4_ measurement techniques are primarily designed for controlled research settings and are not readily scalable to diverse production environments. Thus, there is a need to develop accessible, production-level methods for estimating CH_4_ emissions. This review examines the relationship between enteric CH_4_ emissions and milk fatty acid (FA) composition, highlights key FA groups with potential as biomarkers for indirect CH_4_ estimation, and outlines critical factors of predictive model development. Several milk FAs exhibit strong and consistent correlations to CH_4_ emissions, supporting their utility as predictive biomarkers. Saturated and branched-chain FAs are generally positively associated with CH_4_ emissions, while unsaturated FAs, including linolenic acid, conjugated linoleic acids, and odd-chain FAs, are typically negatively associated. Variability in the strength and direction of correlations across studies is often attributable to differences in diet or lactation stage. Similarly, differences in experimental design, data processing, and model development contribute to much of the variation observed in predictive equations across studies. Future research should aim to (1) identify milk FAs that consistently correlate with CH_4_ emissions regardless of diet, (2) develop robust and standardized prediction models, and (3) prioritize the external validation of prediction models across herds and production systems.

## Simple Summary

Developing accessible methods for measuring methane emissions is essential for the dairy industry to identify inefficiencies and reduce its environmental impact. Current direct measurement techniques are costly, labor-intensive, and largely limited to controlled research environments. An emerging alternative involves analyzing milk fatty acid profiles to estimate emissions, as milk fat synthesis is influenced by the same fermentation pathways in the rumen as methanogenesis. This review investigates the relationship between milk fatty acids and methane emissions, highlights candidates that could serve as biomarkers, and discusses key factors of predictive model development. Saturated fatty acids, especially short-chain and branched-chain acids, exhibit consistent positive relationships with methane emissions across studies, while many unsaturated fatty acids, such as odd-chain and conjugated linoleic and linolenic acids, exhibit consistent negative relationships. Although there are minor discrepancies regarding the strength of these relationships across studies, much of the variation can be attributed to dietary differences and can be corrected in predictive models. Considerable progress has been made in prediction model development; however, inconsistencies in study design and modeling approaches limit their generalizability. Overall, milk fatty acid profiling may offer a promising method for indirect methane estimation, though its utility remains to be confirmed through further research.

## 1. Introduction

**Figure 1:**
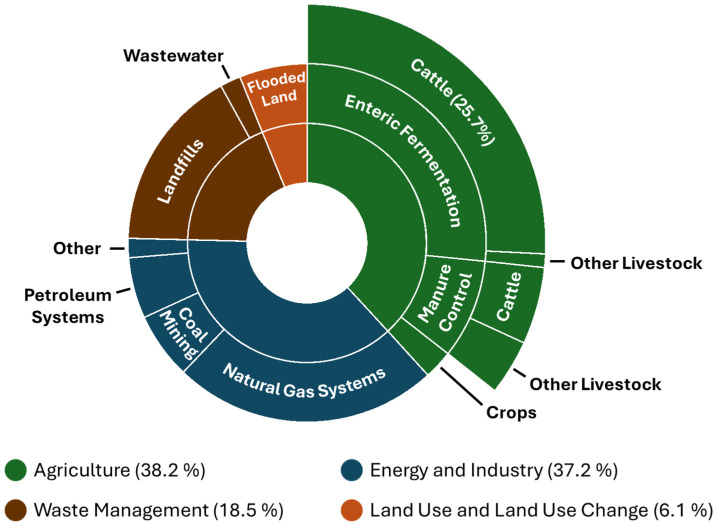
United States anthropogenic methane (CH_4_) emissions by sector and source. Total emissions are categorized into five major sectors: agriculture, energy and industry, waste management, land use and land-use change. The agriculture sector (green) is broken down into three parts: enteric fermentation (26.6% total CH_4_ emissions with cattle making up 97% of those emissions), manure management practices (9% total CH_4_ emissions), and crop cultivation (2.6% total CH_4_ emissions). The energy and industry sector (blue) consists of four parts: natural gas systems, coal mining, petroleum systems, and other activities (23.4, 6.0, 5.5, and 1.8% total CH_4_ emissions, respectively). Landfills and wastewater (16.6 and 1.9% total CH_4_ emissions) represent the major waste management (brown) emission sources. Flooded lands (6.11% total CH_4_ emissions) are the major source of emissions from the land use and land use change sector (orange). Adapted from EPA [3].

Climate change is a global challenge that threatens all ecosystems and poses a threat to production systems across sectors. Agricultural practices contribute significantly to anthropogenic greenhouse gas (GHG) emissions through climate deforestation, land-use change, and enteric fermentation [1]. Methane (CH_4_) emissions are of particular concern because, while short lived, they are extremely efficient at trapping heat in the atmosphere [2]. In the United States, enteric fermentation from livestock is the largest source of anthropogenic CH_4_, accounting for approximately 26% of total emissions (Figure 1) [3]. As the global population rises, the demand for milk and meat products rises in tandem. To meet this growing demand, enhancing livestock production efficiency while reducing CH_4_ emissions is essential for sustainable intensification.

Methane is often referred to as a byproduct of ruminal fermentation, a process by which microbes in the rumen break down complex plant materials, such as fiber and non-fiber carbohydrates, to produce energy for themselves and the host animal [4]. Up to 12% of the gross energy intake is lost to methanogenesis [5]. Thus, reducing CH_4_ emissions could simultaneously enhance feed efficiency and environmental sustainability. Methanogenesis, however, plays a critical role in regulating rumen function. The formation of CH_4_ represents the main pathway for hydrogen (H_2_) removal, a necessary measure for maintaining the redox balance needed for efficient fermentation pathways (reviewed in: Ungerfeld [6]). Suppressing CH_4_ synthesis without redirecting H_2_ towards alternate sinks could impair rumen fermentation, negatively affecting animal health and productivity [7].

To address inefficiencies in feeding or management practices, practical tools for monitoring CH_4_ emissions are required. However, existing CH_4_ measurement technologies are primarily designed for research purposes and have limited feasibility outside controlled environments. An alternative approach involves the indirect estimation of CH_4_ emissions through milk components. Milk fatty acids (FAs), which reflect dietary intake and rumen microbial activity, are already used to measure production parameters [8,9,10]. Because similar biochemical pathways in the rumen influence both methanogenesis and milk fat synthesis, milk FAs may represent potential biomarkers for methanogenesis.

Previous research has evaluated the utility of milk FAs as predictors of CH_4_ emissions, reporting strong correlations between CH_4_ output and several FAs, along with well-performing predictive models. However, important predictors and modeling approaches vary across studies, highlighting the need for standardization and refinement. The objective of this review was to evaluate the potential of milk FAs as biomarkers for enteric CH_4_ emissions in dairy cattle. Specifically, we set out to (1) examine the current evidence linking milk FA to CH_4_ production, (2) identify FAs that consistently correlate with emission levels across studies, and (3) discuss key factors and challenges in the development of robust, generalizable predictive models suitable for practical implementation.

## 2. Greenhouse Gases

Climate change is directly influenced by the amount of GHGs released into the atmosphere [1], and understanding the impact of these gases is critical for developing effective mitigation strategies across global production systems. Greenhouse gases are defined as any gas that traps heat by absorbing infrared radiation [11]. These gases, whether man-made or naturally occurring, build up in the atmosphere and warm the surface of the Earth in a process known as the greenhouse effect [11,12]. Since the Industrial Revolution, atmospheric concentrations of GHGs have increased substantially, largely due to human activities such as the combustion of natural resources (i.e., coal, oil, and gases), agriculture, transportation, and deforestation [1]. Greenhouse gas emissions from these anthropogenic sources have amplified the greenhouse effect, contributing to a rise in global surface temperatures of approximately 0.5 to 1.0 °C over the past century [1,13].

According to the U.S. Environmental Protection Agency (EPA), the major GHGs contributing to climate change are carbon dioxide (CO_2_), CH_4_, nitrous oxide (N_2_O), and fluorinated gases [14]. Each gas is active in the atmosphere for varying amounts of time and has a unique global warming potential (GWP). Global warming potential is used as a standard reference to quantify how much energy one ton of a gas will absorb in the atmosphere relative to one ton of CO_2_ over a specific time period [14]. According to the most recent Assessment Report (AR6 released in 2022) from the Intergovernmental Panel on Climate Change (IPCC), non-fossil CH_4_ has a GWP-20 (i.e., over 20 years) of 79.7 ± 28.5 and a GWP-100 (i.e., over 100 years) of 27.0 ± 11.0, while N_2_O has a GWP-20 of 273 ± 118 and a GWP-100 of 273 ± 130 [2]. These values emphasize the substantial short- and long-term warming contributions of CH_4_ and N_2_O relative to CO_2_, which has a GWP-20 and GWP-100 of one.

## 3. Methane in Agriculture

Agricultural systems contribute approximately 14.5% of global anthropogenic GHG emissions, with cattle production systems accounting for 67% of these emissions [15]. Of the GHGs emitted by agricultural systems, CH_4_ from livestock production systems is given the most focus. In the U.S., enteric fermentation from cattle represents about 26% of total CH_4_ emissions [3]. While CH_4_ has a relatively short atmospheric lifespan (~12.5 years), it is nearly 80 times more potent than CO_2_ over a 20-year period and 27 times more potent over 100 years [2]. Methane emissions have been found to significantly contribute to short-term climate change, and a substantial effort must be made to reduce CH_4_ emissions from agriculture to prevent global temperatures from rising more than 1.5 °C annually [16].

### 3.1. Rumen Methanogenesis

**Figure 2:**
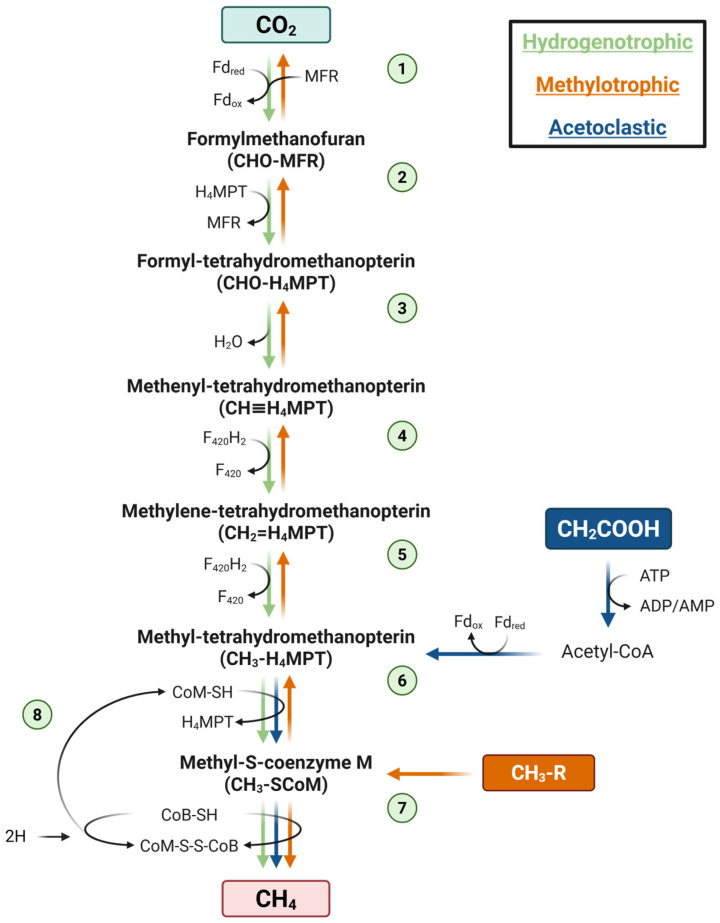
Reactions and enzymes of the three major methanogenic pathways (i.e., hydrogenotrophic, methylotrophic, and acetoclastic) in the rumen. Hydrogenotrophic (green arrows): 1. Free CO_2_ is reduced by ferredoxin (Fd_red_, reduced form; Fd_ox_, oxidized form). Methanofuran (MFR) is attached to the resulting CHO molecule via ligation of formylmethanofuran dehydrogenase. 2. The formyl group is transferred via formylmethanofuran–tetrahydromethanopterin N-formyltransferase to tetrahydromethanopterin (H_4_MPT), forming formyl-H_4_MPT and releasing MFR. 3. Formyl-H_4_MPT is hydrolyzed by N5, N10 methenyltetrahydromethanopterin cyclohydrolase, resulting in methenyl-H_4_MPT and H_2_O. 4. Methenyl-H_4_MPT is reduced to methylene-H_4_MPT by N5, N10-methylenetetrahydromethanopterin dehydrogenase with electrons from coenzyme F_420_. 5. Methylene-H_4_MPT is further reduced to methyl- H_4_MPT by N5, N10-methylenetetrahydromethanopterin reductase with electrons from coenzyme F_420_. 6. H_4_MPT is removed by N5-methyltetrahydromethanopterin, and the methyl group is transferred to coenzyme M (CoM) by coenzyme M-methyltransferase, forming methyl-S-CoM. 7. Reduction via methyl–coenzyme M reductase forms CH_4_, coenzyme B, and coenzyme M (CoB and CoM). 8. Heterodisulfide reductase reduces the sulfur groups on CoB and CoM, which are recycled back into the pathway. Methylotrophic (blue arrows): 1. Methyl groups from methanol/methylamines (CH_3_-R), carried by cognate corrinoid proteins, are transferred to CoM-SH via substrate-specific methyltransferases. 2. In the presence of H_2_, CH_3_-SCoM is reduced to CH_4_, or the lack of H_2_ triggers oxidation of CH_3_-ScoM, subsequently resulting in CO_2_, via the reverse hydrogenotrophic pathway previously described. Acetoclastic (orange arrows): 1. Acetate (CH-COOH) is activated into acetyl-CoA via acetyl-CoA synthetase and ATP. 2. Carbon monoxide dehydrogenase/acetyl-CoA synthase converts acetyl-CoA to methyl and carbonyl groups, then the carbonyl group is oxidized by ferredoxin while the methyl group is transferred to H_4_MPT. 3. CH_4_ is produced following the last two steps of the hydrogenotrophic pathway. Adapted from Lyu et al. [23] and Lessner [24]. Created in BioRender. Youngmark, E. (2025) https://BioRender.com/r19u943 (accessed on 16 May 2025).

In cattle, roughly 87% of enteric CH_4_ is produced in the rumen, with the remaining 13% produced during hindgut fermentation, primarily in the large intestine [17]. Rumen methanogenesis results from the complex interspecies coordination of the rumen microbiota, including bacteria, protists, archaea, and fungi [18,19]. Methanogenic archaea (methanogens) use the end products of fermentation as substrates for growth and CH_4_ production [18]. Three primary pathways are responsible for CH_4_ production in the rumen. The most dominant pathway in adult ruminants is hydrogenotrophic methanogenesis, characterized by the reduction of CO_2_ to form CH_4_ [20]. Acetoclastic and methylotrophic methanogenesis also occur in the rumen in much smaller quantities [20]. Typically, hydrogenotrophic methanogenesis takes place in eight steps starting with CO_2_ as a substrate (Figure 2), though formate can also be used as a precursor [21]. Acetate and methanol/methylamines are used as a substrate for acetoclastic and methylotrophic methanogenesis, respectively [22]. The reduction of each substrate is performed through different processes; however, all pathways share the same final three steps: (1) the methyl group from CH_3_-H_4_MTP is transferred to coenzyme M, (2) coenzyme B facilitates the reduction of coenzyme M, and (3) the CoM-S-S-CoB heterodisulfide is recycled back into the pathway (Figure 2; reviewed in Lyu et al. [23]).

### 3.2. Methanogenesis in Relation to Milk Production

Enteric methanogenesis and milk production are both closely related to rumen function. Most of the energy utilized by ruminants for metabolic processes is derived from volatile fatty acids (VFAs)—the major end products of fermentation in the rumen. Rumen concentrations of the VFAs acetate, butyrate, and propionate are strongly associated with methanogenesis and can be used to predict CH_4_ emissions through simple empirical models [25]. Acetate and butyrate, along with smaller amounts of propionate, serve as key precursors for the *de novo* FA synthesis in the mammary gland (MG) [8,26]. Milk FA composition is further influenced by diet and rumen microbial activity [27,28]. For example, long-chain unsaturated FAs in milk typically originate from the incomplete ruminal biohydrogenation of dietary polyunsaturated fatty acids (PUFAs) [29], while odd- and branched-chain fatty acids (OBCFAs) are synthesized almost exclusively by rumen microbes [27,30].

The strong relationship between milk composition and rumen fermentation has allowed the dairy industry to monitor animal health and productivity for decades [31]. Importantly, milk is readily available and easy to collect, unlike other types of metabolic indicators (i.e., blood and rumen fluid), and its composition is tied to production factors (i.e., age, breed, diet, rumen fermentation efficiencies, stage of lactation, and metabolic processes) [32,33]. Various physiological conditions can be detected or predicted using milk components as biomarkers (i.e., subclinical mastitis [34,35], metabolic and environmental stress [36,37], negative energy balance [9,38], metabolic health [39,40], and fertility [41,42]). In addition to their established applications, milk FAs have been proposed as biomarkers for indirectly estimating CH_4_ emissions from individual animals.

## 4. Methane Measurement Techniques

Techniques for measuring CH_4_ emissions from ruminants date back to at least 1958, when Wainman and Blaxter first described an open-circuit respiration chamber [43]. Since then, many different measurement systems have been developed, all with distinct advantages and limitations depending on research objectives (Table 1). Over the last few decades, respiration chambers (RC), sulfur hexafluoride (SF_6_) tracers, the GreenFeed (GF) and Sniffer systems, hood ventilation, and laser detection have been the most widely adopted techniques to measure CH_4_ emissions from ruminants. While effective for research purposes, these methods present challenges when applied to large-scale production systems. Many are costly to install and maintain and require extensive training for both farm personnel and animals. Additionally, some techniques could be impractical for everyday use based on the need for animals to deviate from typical daily activities.

### 4.1. Respiration Chambers

**Figure 3:**
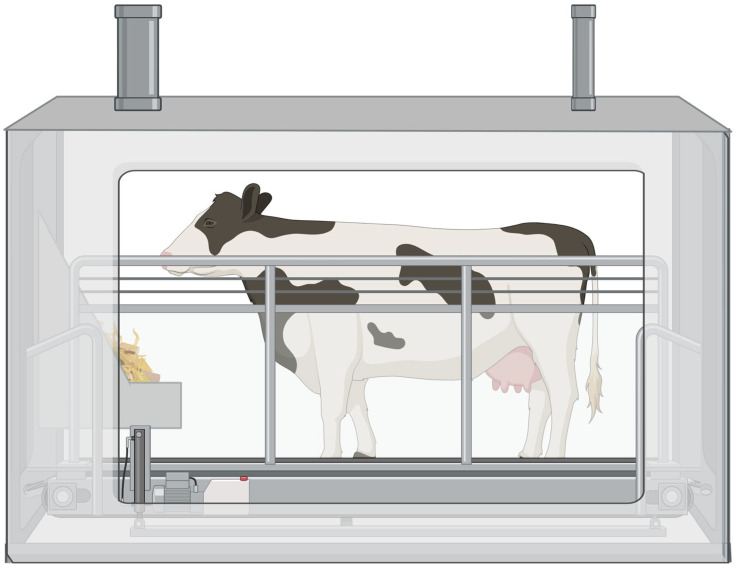
Simplified depiction of methane measurement using a single stall, closed-circuit respiration chamber. This is a sealed, climate-controlled chamber designed to capture gases emitted by a single animal. Ventilation ducts positioned at the top facilitate controlled airflow, enabling accurate measurement of gas concentrations entering and exiting the system. The animal stands on a slatted or solid floor and has access to feed through a mounted trough inside the chamber. Continuous gas sampling allows for precise determination of methane, carbon dioxide, and oxygen. Created in BioRender. Youngmark, E. (2025) https://BioRender.com/1l171×8 (accessed 16 May 2025).

The most robust method of CH_4_ measurement is the respiration chamber (Figure 3). It is commonly referred to as the ‘gold standard’ of emission measurement techniques [5,44]. This technique involves the continuous measurement of CH_4_ emissions from animals housed in an isolated, gas-impermeable chamber. A key advantage of this method is its ability to provide precise, continuous data from both foregut and hindgut emissions [5]. However, animal training is necessary as RCs require partial or total isolation, which can be inherently stressful, subsequently altering metabolism and affecting CH_4_ emissions [44]. Respiration chambers can be designed to accommodate multiple animals at once, but training is still needed to minimize stress. In addition to animal training and labor costs, the construction and upkeep of RCs require substantial expenses [5].

### 4.2. Sulfur Hexafluoride Tracer

**Figure 4:**
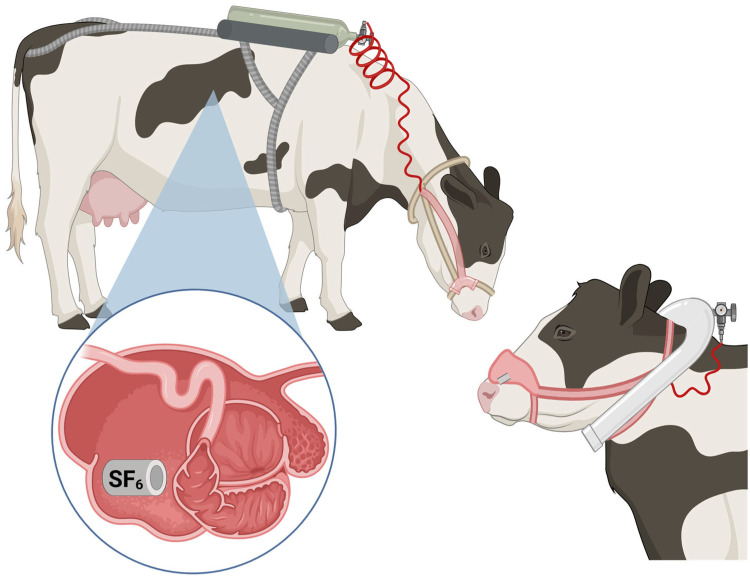
Illustration of a sulfur hexafluoride (SF_6_) tracer bolus and halter equipment. A permeation tube containing the gas tracer is placed into the rumen through the mouth (bottom left) where it releases gas at a known rate. The cow wears a halter containing a sampling apparatus and a nosepiece designed to continuously sample exhaled breath. Gases are collected over a set period and the collection vessel worn on the animal’s back (top left) or as a yolk (bottom right) stores samples until analysis. Created in BioRender. Youngmark, E. (2025) https://BioRender.com/sv564gk (accessed 16 May 2025).

Another widely used method for measuring CH_4_ emissions from ruminants is the sulfur hexafluoride technique (Figure 4). In this technique, patented by Zimmerman [55], a gas tracer (e.g., SF_6_) is orally administered in a permeation tube, and the gas is released at a known rate [5]. The rate at which SF_6_ is released from the mouth and nostrils is measured throughout the day and is subsequently correlated with CH_4_ emission [45]. While less expensive than many other CH_4_ measurement technologies, the SF_6_ technique requires the use of specialized equipment as well as technical expertise to implement [46]. Additionally, permeation tubes require consistent calibration as prolonged use significantly decreases the rate at which SF_6_ is released, resulting in an overestimation of CH_4_ emission [45]. Importantly, SF_6_ is an extremely potent GHG with a GWP-100 of nearly 24,000 [14,56]. This makes large-scale implementation of this CH_4_ measurement technique unsuitable for use as a sustainable application.

### 4.3. GreenFeed System

**Figure 5:**
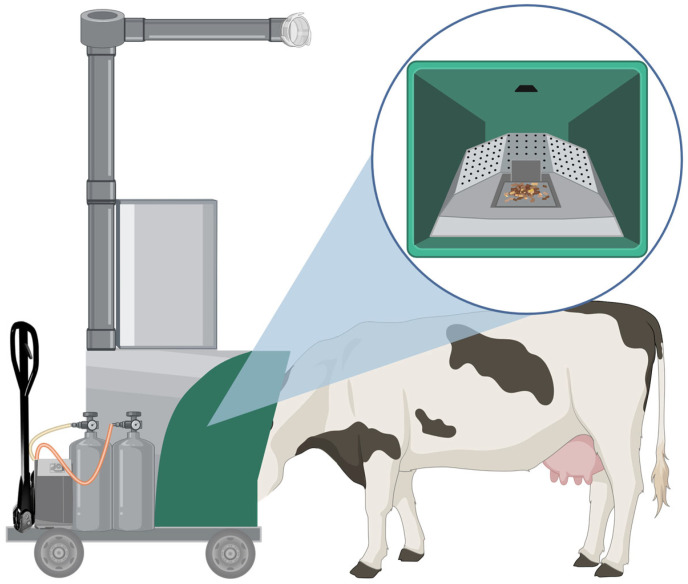
Depiction of a dairy cow using a GreenFeed tie-stall system. The cow is shown standing with its head inserted into the sampling hood of a tie-stall GreenFeed unit. The hood includes an RFID scanner, camera, feed dispenser, and perforated panels with airflow ports to allow the system to identify cows and capture exhaled gases during feeding bouts (highlighted in the inset zoom panel). Above the sampling hood is a feed storage bin and a vertical exhaust and intake duct system, allowing for timed feed release and controlled air movement during sampling. Attached gas cylinders and onboard instrumentation are housed in the body of the unit, enabling real-time analysis of gas concentrations. Created in BioRender. Youngmark, E. (2025) https://BioRender.com/mfhwxud (accessed 15 July 2025).

A relatively recent method of measuring CH_4_ emissions is the GreenFeed (C-Lock™, Rapid City, SD, USA) system (Figure 5). The GF system is a commercially available spot-sampling device that takes continuous gas measurements from a single animal when it is in proximity to the radio frequency identification (RFID) sensor [47]. The GF can be used in all forms of housing, with models designed for pasture, free-stall, and tie-stall production systems. Free-stall and pasture models rely on the voluntary participation of all animals and require training to encourage regular visitation. In contrast, tie-stall models require workers to move the GF to each cow [45]. The tie-stall system provides the most reliable results as the GF unit can be brought to animals at predetermined intervals. When used in pasture or in free-stall settings, however, the data collected may not be representative of the natural CH_4_ production cycle as cows might not visit the GF regularly throughout the day [45].

### 4.4. Sniffer System

**Figure 6:**
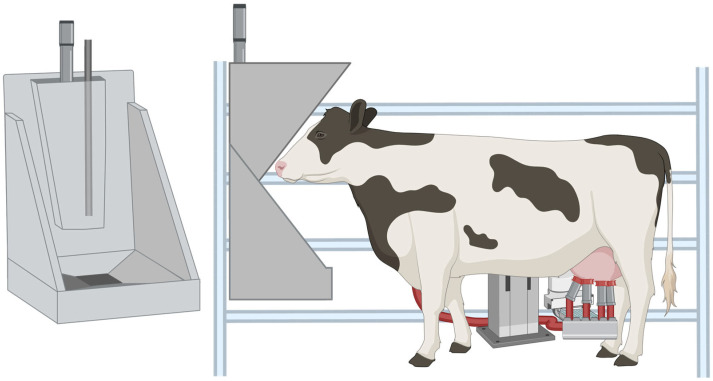
Illustration of methane emission measurement using a Sniffer system in an automated milking unit. The cow is shown standing in an automated milking stall while facing a feed bin outfitted with an overhead gas sampling inlet. Continuous air samples are drawn through a polyethylene tube positioned over the feed bin at the back of the headspace (outlined in the left image) while the cow is eating. Created in BioRender. Youngmark, E. (2025) https://BioRender.com/xae6eje (accessed on 15 July 2025).

The Sniffer method, developed by Garnsworthy et al. [48], was designed as a CH_4_ sampling technique compatible with automated farming systems (Figure 6). In this technique, a polyethylene tube is placed in the feedthroughs of automated milking systems, and gases released through eructation are continuously measured throughout each milking [48]. It enables repeated, individual methane measurements from a large number of animals each day, with minimal equipment and limited need for additional animal training. Like other spot-sampling systems, the Sniffer method does not measure total CH_4_ production. Instead, it predicts daily emissions using regression equations [48]. One disadvantage to the Sniffer method is its relatively low accuracy and considerable variation between and within cows [49,57].

### 4.5. Stoichiometric Approaches to Estimating Enteric Methane Emissions

Indirect CH_4_ measurement techniques have also been widely used in research, as they are generally less expensive and require less specialized equipment. The stoichiometric relationship between enteric methanogenesis and ruminal VFA production is well-established in the literature [54,58]. These methods predict CH_4_ output by applying measured or predicted molar proportions, or the molar production, of ruminal VFAs to stochiometric models [8,59,60]. Although these models have improved greatly since their inception, they still exhibit inconsistences in predicting emissions and require further refinement [25]. Additionally, the collection of rumen contents, a prerequisite for this method, is invasive and requires skilled labor, further limiting its large-scale application.

## 5. Milk Fatty Acids as Biomarkers

### 5.1. Origin of Milk Fatty Acids and Their Relationship to Methane

Bovine milk fat is a complex matrix comprising at least 400 individual FAs [61]. Approximately 97–98% of the FAs found in bovine milk are bound to triacylglycerides, with the remaining 2–3% found in diacylglycerols, monoacylglycerols, phospholipids, and cholesterols or as free FAs [62]. Milk FAs originate from two major sources: (i) *de novo* synthesis in the MG and (ii) performed FAs (either from the diet, endogenous and microbial metabolism, or microbial membranes) [8,28]. Fatty acids derived from *de novo* synthesis are typically saturated and between 4 and 14 carbons in length. However, half of all 16:0 and small amounts of odd- and branched-chain FAs (OBCFAs) are also produced *de novo* in the MG [60,62]. Conversely, most long-chain FAs (LCFA; ≥14 carbons), OBCFAs, and unsaturated FAs in milk are delivered to the MG preformed. For example, milk OBCFAs are synthesized by rumen microbes, primarily as structural components of their cellular membranes [8], while LCFAs and unsaturated FAs (UFAs) are formed through the biohydrogenation or mammary desaturation of dietary polyunsaturated FAs (PUFAs). These include half of 16:0, nearly all of 18:0, and a large portion of 18:1 and 18:2 isomers [62,63].

The milk FA profile is highly responsive to dietary manipulation and rumen fermentation [64,65]. Specifically, the synthesis and uptake of FAs by the MG are influenced by rumen VFA proportions, fermentation patterns, and microbial activity [8,66]. Acetate and butyrate, the primary substrates for *de novo* FA synthesis in the MG, are positively associated with CH_4_ emissions [25]. During microbial fermentation, the production of acetate and butyrate results in a net release of H_2_, promoting the activity of methanogens and increasing the rate of methanogenesis [7]. Conversely, propionate, a crucial substrate for gluconeogenesis and a precursor for OBCFA synthesis in the rumen and MG, is negatively associated with CH_4_ emissions [8]. Propionate synthesis requires the consumption of H_2_, thereby acting as a competitive pathway that limits methanogenesis [7]. Due to the stoichiometric relationship between molar VFA proportions and methanogenesis in the rumen, and the strong functional relationship between VFAs leaving the rumen and milk FA synthesis, a close connection can be assumed between the milk FA profile and rumen methanogenesis.

### 5.2. Saturated Fatty Acids

Saturated fatty acids (SFA) make up approximately 68% of all milk FAs and are predominantly produced *de novo* in the MG [67]. Increased proportions of SFAs in milk are widely assumed to correlate with increased CH_4_ emissions, as their synthesis is known to increase with the rumen proportions of acetate and butyrate. This relationship between SFA milk content and CH_4_ emission parameters has been repeatedly observed across multiple studies [68,69,70]. Overall, SFAs as a group have a positive relationship with CH_4_ emissions, though reported correlation strengths vary across studies (Supplementary Tables S1 and S2).

Dietary variation likely accounts for the inconsistences observed between studies. Diets fed to cows in each study or dataset were experimental and designed to reduce CH_4_ production in the rumen though the supplementation of myristic acid (14:0) [68], linseed or linseed oil [69,70], or a combination of supplements from external studies [71,72]. The dietary interventions likely altered rumen fermentation patterns, microbial community structure and activity, and methanogenesis in unique ways, thereby impacting milk FA synthesis [60,62].

### 5.3. Unsaturated Fatty Acids

Unsaturated FAs make up approximately one-third of the total milk fat, with monounsaturated fatty acids (MUFAs) accounting for about 27% and PUFAs comprising 4% [73]. As a group, UFAs, as well as many individual MUFAs and PUFAs, exhibit negative relationships with CH_4_ emission parameters [70,71,72]. This relationship is expected, as it aligns with the established mechanisms of rumen lipid metabolism. Generally, *trans*-MUFAs and *trans*-PUFAs (i.e., *trans* isomers of 16:1, 18:1, and 18:2) are derived from the incomplete biohydrogenation of dietary lipids, and an increase in these FAs in milk would reflect a higher dietary fat intake [74]. Similarly, 18:1 *c*9 is associated with increased lipid supplementation, as a higher dietary intake leads to a greater ruminal outflow of 18:1 *c*9, in addition to a greater supply of 18:0 serving as a substrate for Δ^9^-desaturase-mediated conversion in the MG [74]. Increased lipid intake is known to suppress rumen methanogenesis by inhibiting fiber degradation within the rumen [69,74]. Importantly, lipid supplementation and forage composition alter the milk FA profile. High forage diets and lipid supplements (e.g., linseed or linseed oil) commonly used in studies to mitigate CH_4_ emissions, alter the rumen FA metabolism and thereby affect the abundance of biohydrogenation intermediates and end products in milk (e.g., 18:1 and 18:2 isomers) [70,74,75].

A study by Chilliard et al. [69] reported that the milk FAs exhibiting the strongest negative correlations with CH_4_ emissions were biohydrogenation intermediates of α-linolenic acid (e.g., 18:1 *t*6 + 7 + 8; 18:1 *t*12; 18:1 *t*13 + 14; 18:1 *t*16; 18:2 *c*9,*t*13; 18:1 *c*15; and 18:2 *t*11,*c*15). Additionally, strong negative correlations were observed for total C18 FAs, 18:1-*cis* isomers, and 18:1-*trans* isomers [69]. These findings are largely supported by subsequent studies [60,63,64,70], which also report predominantly moderate to strong negative associations between 18:1, 18:2, and 18:3 isomers and CH_4_ emission parameters. However, the strength and direction of these correlations varied across studies (Supplementary Tables S1 and S2), likely as a consequence of differing dietary interventions (i.e., fat type, oilseed inclusion, and forage composition), and careful interpretation is necessary when analyzing these FAs.

### 5.4. Odd- and Branched-Chain Fatty Acids

Odd- and branched-chain FAs comprise up to 3% of the total FAs in ruminant milk fat [68]. The rumen microbial synthesis of OBCFAs is thought to depend more on microbial FA synthase activity than substate availability [74]. Therefore, milk OBCFA profiles are reflective of rumen microbial activity and have been proposed as biomarkers of rumen function [66]. Several OBCFAs have been identified as predictors of rumen VFA concentrations, and by extension, CH_4_ emissions, due to the stoichiometric relationship between VFA production and CH_4_ formation in the rumen [8,66,76].

The most characteristic OCFAs found in ruminant milk are pentadecanoic acid (15:0), heptadecanoic acid (17:0), and heptadecenoic acid (17:1 *c*9) [77]. Several studies have reported positive correlations between the OCFAs 15:0 and 17:0 and ruminal propionate proportions, indicating an inverse relationship with CH_4_ production [60,66,78]. However, other studies have observed positive associations, or no associations, between these OBCFAs and CH_4_ emissions [69,70,71,72], indicating an inconsistency across findings (Supplementary Tables S1 and S2). In contrast, strong negative correlations between CH_4_ emission parameters and 17:1 *c*9, as well as the sum of 17:0 and 17:1 *c*9, have been consistently reported across studies [60,70,71,79]. Discrepancies in the association of 15:0 and 17:0 with CH_4_ parameters may be attributed to the endogenous synthesis of these FAs in the small intestine or MG, particularly in animals supplemented with linseed oil, which may inhibit *de novo* microbial FA synthesis in the rumen [60,69,79].

The branched-chain FAs (BCFAs) 13:0-*iso*, 14:0-*iso*, 15:0-*iso*, 16:0-*iso*, 17:0-*iso*, 18:0-*iso*, 13:0-*anteiso*, 15:0-*anteiso*, and 17:0-*anteiso* are primarily synthesized by rumen microbes via the elongation of branched-chain amino acids, specifically valine, leucine, and isoleucine [80,81]. These amino acids serve as precursors for the formation of *iso*- and *anteiso* FA structures, which are incorporated into microbial membranes and can subsequently appear in milk fat [82]. Milk 14:0-*iso* and 15:0-*iso* were identified as strong predictors of rumen VFA proportions, particularly exhibiting positive correlations with acetate, suggesting an indirect association with CH_4_ production [8,66]. This relationship is further supported in later studies, which reported positive correlations not only for 14:0-*iso* and 15:0-*iso*, but also for 15:0-*anteiso*, 16:0-*iso*, and 17:0-*anteiso* [60,71,72].

Conversely, van Gastelen et al. [83] found no overall relationship between *iso*- or *anteiso*-BCFA and CH_4_ production, although the authors describe a positive association between 15:0-*iso* and CH_4_ yield (g/kg dry matter intake (DMI)) and intensity (g/kg fat- and protein-corrected milk (FPCM)), and a negative association between 14:0-*iso* and CH_4_ yield. Additionally, Engelke et al. [70] observed CH_4_ production negatively correlated with 15:0-*iso*, and weak to no correlations for 14:0-*iso*, 15:0-*anteiso*, 16:0-*iso*, and 17:0-*anteiso*. Differences in correlation strength and direction between these studies may be explained by dietary influences on rumen microbial communities. *iso*-BCFAs are predominately associated with cellulolytic bacteria, whereas *anteiso*-BCFAs are linked to amylolytic bacteria [81]. Variations in forage to concentrate ratios, forage type and quality, oil supplementation, and crude protein concentrations can alter the bacterial populations and activity, thereby affecting microbial FA synthesis and the milk FA profile [72,84].

## 6. Development of Prediction Models

The use of milk FA profiles to predict CH_4_ emissions and rumen fermentation patterns has been explored for decades [8,70]. Despite the growing body of literature aimed at refining existing models and developing new ones, substantial discrepancies remain between studies [69,70,79]. Comparisons across models are challenging, as differences in experimental design and statistical modeling approach limit the generalizability of findings [85]. Several meta-analyses have tackled the challenge of compiling data from individual trials to compare the proposed models and determine the most reliable FA predictors [85,86,87]. The analyses of Castro-Montoya et al. [85], van Lingen et al. [86], and Bougouin et al. [87] report a moderate potential for milk FAs to predict CH_4_ emission, but their conclusions differ. Castro-Montoya et al. [85] reported that 16:1 and 18:1 isomers could serve as effective predictors of CH_4_ output, while van Lingen et al. [86] found that some SFAs and 18:1 isomers were reasonably predictive of CH_4_ yield and intensity, though no strong relationships with CH_4_ output were established. In contrast, Bougouin et al. [87] observed that prediction models only using FA variables were outperformed by those that incorporated production parameters (e.g., DMI, days in milk, body weight, and dietary intake of nutrients). These conflicting results highlight the challenges of building robust predictive models from highly variable datasets.

### 6.1. Data Processing and Variable Selection

Methane emission metrics, such as CH_4_ output, yield, and intensity, are commonly reported in g/d, g/kg DMI, and g/kg FPCM, respectively [71,76,86]. However, these are not standardized units and can vary greatly between studies. Some studies use VFA concentration to predict CH_4_ emissions and report estimated CH_4_ emissions as a molar proportion (e.g., mmol CH_4_/mol VFA) [60,78]. Similarly, milk FA proportions are expressed using a range of unstandardized units. Most publications report values in g/100 g of total FA [60,69,88], but they are also commonly reported as a percentage of total milk fat or total fatty acid methyl esters (FAMEs) [70,79].

High dimensionality is addressed in some studies by grouping FAs by saturation class (e.g., SFAs, MUFAs, and PUFAs) or carbon chain length, although most include a combination of grouped and individual FAs [70,72,85]. Dimensionality is further reduced by omitting minor FAs with limited variance, but the criteria for exclusion vary and are not always well-justified. For example, some studies do not exclude any FAs, while others define “minor” FAs as those present at <0.1% g/100 g total FA [85,87], <0.02% g/100 g total FA [69], or <0.02% total FAMEs [79].

Given the inherent multicollinearity among FAs due to shared metabolic pathways, variable selection is a critical step. Most studies use univariate filtering to exclude highly collinear or weakly correlated variables, followed by stepwise regression using selection criteria such as Akaike information criterion (AIC) [72,83] or Bayesian information criterion (BIC) [71,87] to retain only the most robust variables. Some models emphasize biological relevance as a selection criterion, prioritizing FAs derived from rumen microbial metabolism (e.g., OBCFAs) [60,66]. Production parameters, such as DMI, energy-corrected milk, and fat intake, are also commonly included as explanatory variables.

### 6.2. Modeling Approaches

Multiple linear regression remains the most widely used technique for modeling CH_4_ emissions based on milk FA profiles [69,83,88]. Simple linear regression models typically use CH_4_ output, yield, or intensity as the dependent variable, with selected FAs and production parameters as independent variables. When repeated measures are available or multiple dietary treatments are used, mixed-effects models are often applied to account for the random effects associated with animal, diet, or time [86,89]. This step improves model generalizability by reducing bias and accounting for individual variation. While less common, partial least squares regression has been employed to address multicollinearity in the dataset, as it yields latent variables that maximize covariance between FA and CH_4_ outcomes [60,66].

### 6.3. Model Evaluation

Model performance is commonly assessed using standard fit statistics such as the coefficient of determination (R^2^), root mean squared error (RMSE), root mean square prediction error (RMSPE), concordance correlation coefficient (CCC), AIC, and BIC [60,66,83]. Cross-validation techniques, especially k-fold and leave-one-out methods, are widely used to prevent overfitting [60,78,87]. While some studies incorporate equations from previously published models [88,89], independent external validation remains rare. Furthermore, model checks such as residual plots, variance inflation factors, and tests for normality or heteroscedasticity are reported inconsistently, which limits reproducibility and critical evaluation.

Collectively, these modeling strategies demonstrate the potential of milk FA profiles as predictive tools for CH_4_ emissions. However, key limitations persist. Many datasets are often small relative to the number of predictors, and multicollinearity is not always adequately addressed. Most importantly, a lack of model validation on independent populations is not widely performed, leaving many models narrowly tailored to specific diets, breeds, or production types. Broader model applicability will require standardized methods, larger and more diverse datasets, and consistent validation across independent populations.

## 7. Conclusions

Milk is a readily available biological matrix that offers valuable opportunities to investigate indicators of animal health, production, and environmental impact. A variety of milk FAs have been proposed as biomarkers of rumen function and CH_4_ production, with many demonstrating direct associations with rumen function. Among the most consistently reported FAs are short-chain SFAs, 18:1 and 18:2 isomers, *iso*-BCFAs, and 17:1 *c*9. Nonetheless, future research is needed to refine the selection of FAs best suited for predictive modeling. While substantial progress has been made in developing models that relate milk FAs to CH_4_ emissions, refinement is still needed to improve their reliability and applicability. This approach remains exploratory and requires substantial further research before any practical application can be recommended. Priority should be given to enhancing variable selection, optimizing model structure, and implementing rigorous cross-validation to ensure models meet both the scientific standards and operational needs of sustainable dairy production. With continued advancement, predictive models based on milk FAs may provide a practical and scalable solution for estimating CH_4_ emissions and supporting climate-resilient dairy systems.

## Figures and Tables

**Table 1:** List of common methane measurement techniques commonly used in research, and the pros and cons associated with their large-scale application.

CH_4_ Measurement Technique	Pros	Cons	References
Respiration chamber	‘Gold standard’ High accuracy Continuous measurement of foregut and hindgut emissions	High cost for installation and upkeep Labor intensive Animal isolation Limited capacity	[5,44]
Sulfur hexafluoride (SF_6_) tracer	Non-restrictive Can be adapted to all production types	Invasive Labor and equipment Unsustainable (GWP-100 of SF_6_ is 24,000)	[3,5,45]
GreenFeed	Non-invasive Can be adapted to all production types	High cost (installation and feed) Labor intensive Limited capacity Challenges in capturing absolute emission values	[46,47]
Sniffer	Low cost Non-invasive Large capacity	High measurement variability Limited to robotic dairies	[48,49]
Laser detection	Low cost Non-invasive Easy to use handheld device	Labor intensive Accuracy limited by field conditions	[50,51]
CH_4_/CO_2_ ratio	Low cost Non-invasive No equipment needed	Estimation of both CO_2_ and CH_4_ emissions High measurement variability	[52,53]
VFA measurement	Low cost Well-established	Invasive Labor intensive Requires specialized equipment	[25,54]

CH_4_, methane; CO_2_, carbon dioxide; GWP-100, global warming potential over 100 years; SF_6_, sulfur hexafluoride; and VFA, volatile fatty acid

## Data Availability

Not applicable.

## Supplementary Materials

The following supporting information can be downloaded at: https://www.mdpi.com/article/10.3390/ani15152212/s1, Table S1: Summary of reported correlations between milk fatty acids (FA) and methane (CH_4_) emission metrics and their reported strengths across published studies, including whether the association was positive (+), negative (-), or absent (Ø), and the strength of the correlation (W = weak, M = moderate, S = strong); Table S2: Reported correlations between milk fatty acids (FA, g/100 g total FA) and methane (CH_4_) emission metrics (Output, g/d; Yield, g/kg dry matter intake; Intensity, g/kg milk unit) across studies. Ref. [90] is cited in Supplementary Materials.

## Abbreviations

The following abbreviations are used in this manuscript:

AIC	Akaike information criterion
BCFA	Branched-chain fatty acid
BHBA	Beta-hydroxybutyrate
BIC	Bayesian information criterion
CCC	Concordance correlation coefficient
CH_3_	Methyl
CH_4_	Methane
CH_2_COOH	Acetate
CLA	Conjugated linoleic acid
CO_2_	Carbon dioxide
CoM	Coenzyme M
CoB	Coenzyme B
EPA	Environmental Protection Agency
FA	Fatty acid
Fd_ox_	Ferredoxin-oxidized
Fd_red_	Ferredoxin-reduced
GF	GreenFeed
GHG	Greenhouse gas
GWP	Global warming potential
H_2_	(Di) hydrogen
H_2_O	Water
H_4_MPT	Tetrahydromethanopterin
IPCC	Intergovernmental Panel on Climate Change
LCFA	Long-chain fatty acid
MFR	Methanofuran
MG	Mammary gland
MUFA	Monounsaturated fatty acid
N_2_O	Nitrous oxide
OBCFA	Odd- and branched-chain fatty acid
OCFA	Odd-chain fatty acid
PUFA	Polyunsaturated fatty acid
RC	Respiration chamber
RFID	Radio-frequency identification
RMSE	Root mean squared error
RMSPE	Root mean squared prediction error
SF_6_	Sulfur hexafluoride
SFA	Saturated fatty acid
SMCFA	Short- and medium-chain fatty acid
UFA	Unsaturated fatty acid
VFA	Volatile fatty acid

## References

[1] Mikhaykov A., Moiseev N., Aleshin K., Burkhardt T. (2020). Global climate change and greenhouse effect. Entrep. Sustain. Issues.

[2] IPCC (2021). Climate Change 2021: The Physical Science Basis.

[3] EPA (2024). Inventory of U.S. Greenhouse Gas Emissions and Sinks: 1990–2022 U.S. Environmental Protection Agency. EPA 430R-24004. https://www.epa.gov/ghgemissions/inventory-us-greenhouse-gas-emissions-and-sinks-1990-2022.

[4] Russell J.B., Hespell R.B. (1981). Microbial rumen fermentation. J. Dairy Sci..

[5] Johnson K.A., Johnson D.E. (1995). Methane emissions from cattle. J. Anim. Sci..

[6] Ungerfeld E.M. (2020). Metabolic hydrogen flows in rumen fermentation: Principles and possibilities of interventions. Front. Microbiol..

[7] Ungerfeld E.M. (2015). Shifts in metabolic hydrogen sinks in the methanogenesis-inhibited ruminal fermentation: A meta-analysis. Front. Microbiol..

[8] Vlaeminck B., Fievez V., Tamminga S., Dewhurst R.J., van Vuuren A., De Brabander D., Demeyer D. (2006). Milk odd- and branched-chain fatty acids in relation to the rumen fermentation pattern. J. Dairy Sci..

[9] Churakov M., Karlsson J., Edvardsson Rasmussen A., Holtenius K. (2021). Milk fatty acids as indicators of negative energy balance of dairy cows in early lactation. Animals.

[10] Angulo J., Hiller B., Olivera M., Mahecha L., Dannenberger D., Nuernberg G., Losand B., Nuernberg K. (2012). Dietary fatty acid intervention of lactating cows simultaneously affects lipid profiles of meat and milk. J. Sci. Food Agric..

[11] Tuckett R., Worsfold P., Poole C., Townshend A., Miró M. (2019). Greenhouse gases. Encyclopedia of Analytical Science.

[12] Raval A., Ramanathan V. (1989). Observational determination of the greenhouse effect. Nature.

[13] Ramanathan V., Cicerone R.J., Singh H.B., Kiehl J.T. (1985). Trace gas trends and their potential role in climate change. J. Geophys. Res. Atmos..

[14] EPA U.S. Overview of Greenhouse Gases. https://www.epa.gov/ghgemissions/overview-greenhouse-gases.

[15] AGA, Food and Agriculture Organization of the United Nations (2017). Livestock Solutions for Climate Change.

[16] Arndt C., Hristov A.N., Price W.J., McClelland S.C., Pelaez A.M., Cueva S.F., Oh J., Dijkstra J., Bannink A., Bayat A.R. (2022). Full adoption of the most effective strategies to mitigate methane emissions by ruminants can help meet the 1.5 degrees C target by 2030 but not 2050. Proc. Natl. Acad. Sci. USA.

[17] Ellis J.L., Dijkstra J., Kebreab E., Bannink A., Odongo N.E., McBride B.W., France J. (2008). Aspects of rumen microbiology central to mechanistic modelling of methane production in cattle. J. Agric. Sci..

[18] Wolin M.J., McDonald I.W., Warner A.C.I. (1975). Interactions between the bacterial species of the rumen. Digestion & Metabolism in the Ruminant.

[19] Martínez-Álvaro M., Auffret M.D., Duthie C.-A., Dewhurst R.J., Cleveland M.A., Watson M., Roehe R. (2022). Bovine host genome acts on rumen microbiome function linked to methane emissions. Commun. Biol..

[20] Greening C., Geier R., Wang C., Woods L.C., Morales S.E., McDonald M.J., Rushton-Green R., Morgan X.C., Koike S., Leahy S.C. (2019). Diverse hydrogen production and consumption pathways influence methane production in ruminants. ISME J..

[21] Leahy S.C., Kelly W.J., Altermann E., Ronimus R.S., Yeoman C.J., Pacheco D.M., Li D., Kong Z., McTavish S., Sang C. (2010). The genome sequence of the rumen methanogen methanobrevibacter ruminantium reveals new possibilities for controlling ruminant methane emissions. PLoS ONE.

[22] Friedman N., Jami E., Mizrahi I. (2017). Compositional and functional dynamics of the bovine rumen methanogenic community across different developmental stages. Environ. Microbiol..

[23] Lyu Z., Shao N., Akinyemi T., Whitman W.B. (2018). Methanogenesis. Curr. Biol..

[24] Lessner D.J. (2009). Methanogenesis biochemistry. Encyclopedia of Life Sciences.

[25] Williams S.R.O., Hannah M.C., Jacobs J.L., Wales W.J., Moate P.J. (2019). Volatile fatty acids in ruminal fluid can be used to predict methane yield of dairy cows. Animals.

[26] Wang B., Mao S.Y., Yang H.J., Wu Y.M., Wang J.K., Li S.L., Shen Z.M., Liu J.X. (2014). Effects of alfalfa and cereal straw as a forage source on nutrient digestibility and lactation performance in lactating dairy cows. J. Dairy Sci..

[27] Lou Z., Evans A.C.O., Bu D. (2024). The relation and variation of OBCFA content in rumen fluid, blood and milk from lactating dairy cows. Livest. Sci..

[28] Kliem K.E., Humphries D.J., Kirton P., Givens D.I., Reynolds C.K. (2019). Differential effects of oilseed supplements on methane production and milk fatty acid concentrations in dairy cows. Animal.

[29] Collomb M., Schmid A., Seieber R., Wechsler D., Ryhänen E. (2006). Conjugated linoleic acids in milk fat: Variation and physiological effects. Int. Dairy J..

[30] Bainbridge M.L., Cersosimo L.M., Wright A.D., Kraft J. (2016). Content and composition of branched-chain fatty acids in bovine milk are affected by lactation stage and breed of dairy cow. PLoS ONE.

[31] Krogh M.A., Hostens M., Salavati M., Grelet C., Sorensen M.T., Wathes D.C., Ferris C.P., Marchitelli C., Signorelli F., Napolitano F. (2020). Between- and within-herd variation in blood and milk biomarkers in Holstein cows in early lactation. Animal.

[32] Stoop W.M., Bovenhuis H., Heck J.M.L., van Arendonk J.A.M. (2009). Effect of lactation stage and energy status on milk fat composition of Holstein-Friesian cows. J. Dairy Sci..

[33] Carroll S.M., DePeters E.J., Taylor S.J., Rosenberg M., Perez-Monti H., Capps V.A. (2006). Milk composition of Holstein, Jersey, and Brown Swiss cows in response to increasing levels of dietary fat. Anim. Feed Sci. Technol..

[34] Antanaitis R., Juozaitiene V., Jonike V., Baumgartner W., Paulauskas A. (2021). Milk lactose as a biomarker of subclinical mastitis in dairy cows. Animals.

[35] Hussein H.A., El-Razik K., Gomaa A.M., Elbayoumy M.K., Abdelrahman K.A., Hosein H.I. (2018). Milk amyloid a as a biomarker for diagnosis of subclinical mastitis in cattle. Vet. World.

[36] Garro-Aguilar Y., Fernandez R., Calero S., Noskova E., Gulak M., de la Fuente M., Adell A., Simon E., Muzquiz U., Rodriguez-Pinon D. (2023). Acute stress-induced changes in the lipid composition of cow’s milk in healthy and pathological animals. Molecules.

[37] Cai-yun F., Di S., He T., Rui-ting H., Lei R., Ying Y., Yan-jing S., Jian-bo C. (2019). Milk production and composition and metabolic alterations in the mammary gland of heat-stressed lactating dairy cows. J. Integr. Agric..

[38] Delosière M., Pires J., Bernard L., Cassar-Malek I., Bonnet M. (2019). Milk proteome from in silico data aggregation allows the identification of putative biomarkers of negative energy balance in dairy cows. Sci. Rep..

[39] Tessari R., Mazzotta E., Blasi F., Morgante M., Badon T., Bedin S., Fabbri G., Lisuzzo A., Contiero B., Fiore E. (2021). Milk fatty acids as biomarkers of metabolic diseases in dairy cows identified through thin layer chromatography and gas chromatographic techniques (tlc-gc). Large Anim. Rev..

[40] Heirbaut S., Jing X.P., Stefanska B., Pruszynska-Oszmalek E., Buysse L., Lutakome P., Zhang M.Q., Thys M., Vandaele L., Fievez V. (2023). Diagnostic milk biomarkers for predicting the metabolic health status of dairy cattle during early lactation. J. Dairy Sci..

[41] Menezes C., Malo-Estepa I., Johnston D., Delaney A., Crowe M., Diskin M., Dempsey E. (2020). Electrochemical assay of sorbitol dehydrogenase at pedot modified electrodes—A new milk biomarker for confirmation of pregnancy in dairy cattle. Analyst.

[42] Ntallaris T., Bage R., Karlsson J., Holtenius K. (2023). Milk fatty acids as indicators of delayed commencement of luteal activity in dairy cows in early lactation. Reprod. Domest. Anim..

[43] Blaxter K.L., Clapperton J.L. (1965). Prediction of the amount of methane produced by ruminants. Br. J. Nutr..

[44] Llonch P., Troy S.M., Duthie C., Somarriba M., Rooke J., Haskell M.J., Rainer R., Turner S.P. (2018). Changes in feed intake during isolation stress in respiration chambers may impact methane emissions assessment. Anim. Prod. Sci..

[45] Deighton M.H., O’Loughlin B.M., Williams S.R.O., Moate P.J., Kennedy E., Boland T.M., Eckard R.J. (2013). Declining sulphur hexafluoride permeability of polytetrafluoroethylene membranes causes overestimation of calculated ruminant methane emissions using the tracer technique. Anim. Feed Sci. Technol..

[46] Hristov A.N., Oh J., Giallongo F., Frederick T., Weeks H., Zimmerman P.R., Harper M.T., Hristova R.A., Zimmerman R.S., Branco A.F. (2015). The use of an automated system (GreenFeed) to monitor enteric methane and carbon dioxide emissions from ruminant animals. J. Vis. Exp..

[47] McGinn S.M., Coulombe J.F., Beauchemin K.A. (2021). Technical note: Validation of the GreenFeed system for measuring enteric gas emissions from cattle. J. Anim. Sci..

[48] Garnsworthy P.C., Craigon J., Hernandez-Medrano J.H., Saunders N. (2012). On-farm methane measurements during milking correlate with total methane production by individual dairy cows. J. Dairy Sci..

[49] Bell M.J., Potterton S.L., Craigon J., Saunders N., Wilcox R.H., Hunter M., Goodman J.R., Garnsworthy P.C. (2014). Variation in enteric methane emissions among cows on commercial dairy farms. Animal.

[50] Sorg D., Difford G.F., Mühlbach S., Kuhla B., Swalve H.H., Lassen J., Strabel T., Pszczola M. (2018). Comparison of a laser methane detector with the greenfeed and two breath analysers for on-farm measurements of methane emissions from dairy cows. Comput. Electron. Agric..

[51] Sorg D. (2022). Measuring livestock CH_4_ emissions with the laser methane detector: A review. Methane.

[52] Madsen J., Bjerg B.S., Hvelplund T., Weisbjerg M.R., Lund P. (2010). Methane and carbon dioxide ratio in excreted air for quantification of the methane production from ruminants. Livest. Sci..

[53] Suzuki T., Kamiya Y., Oikawa K., Nonaka I., Shinkai T., Terada F., Obitsu T. (2021). Prediction of enteric methane emissions from lactating cows using methane to carbon dioxide ratio in the breath. Anim. Sci. J..

[54] Leng R.A., Phillipson A.T. (1970). Formation and production of volatile fatty acids in the rumen. Physiology of Digestion and Metabolism in the Ruminant.

[55] Zimmerman P.R. (1993). System for Measuring Metabolic Gas Emissions from Animals. No.

[56] Dervos C.T., Vassiliou P. (2000). Sulfur hexafluoride (SF_6_): Global environmental effects and toxic byproduct formation. J. Air Waste Manag. Assoc..

[57] Garnsworthy P.C., Craigon J., Hernandez-Medrano J.H., Saunders N. (2012). Variation among individual dairy cows in methane measurements made on farm during milking. J. Dairy Sci..

[58] Demeyer D.I., Van Nevel C.J., McDonald I.W., Warner A.C.I. (1975). Methanogenesis, an integrated part of carbohydrate fermentation, and its control. Digestion and Metabolism in the Ruminant.

[59] Alemu A.W., Dijkstra J., Bannink A., France J., Kebreab E. (2011). Rumen stoichiometric models and their contribution and challenges in predicting enteric methane production. Anim. Feed Sci. Technol..

[60] Castro-Montoya J., Bhagwat A.M., Peiren N., De Campeneere S., De Baets B., Fievez V. (2011). Relationships between odd- and branched-chain fatty acid profiles in milk and calculated enteric methane proportion for lactating dairy cattle. Anim. Feed Sci. Technol..

[61] Jensen R.G. (2002). The composition of bovine milk lipids: January 1995 to December 2000. J. Dairy Sci..

[62] Parodi P.W. (2004). Milk fat in human nutrition. Aust. J. Dairy Technol..

[63] Glasser F., Ferlay A., Doreau M., Schmidely P., Sauvant D., Chilliard Y. (2008). Long-chain fatty acid metabolism in dairy cows: A meta-analysis of milk fatty acid yield in relation to duodenal flows and de novo synthesis. J. Dairy Sci..

[64] Livingstone K.M., Humphries D.J., Kirton P., Kliem K.E., Givens D.I., Reynolds C.K. (2015). Effects of forage type and extruded linseed supplementation on methane production and milk fatty acid composition of lactating dairy cows. J. Dairy Sci..

[65] Bayat A.R., Kairenius P., Stefanski T., Leskinen H., Comtet-Marre S., Forano E., Chaucheyras-Durand F., Shingfield K.J. (2015). Effect of camelina oil or live yeasts (*Saccharomyces cerevisiae*) on ruminal methane production, rumen fermentation, and milk fatty acid composition in lactating cows fed grass silage diets. J. Dairy Sci..

[66] Fievez V., Colman E., Castro-Montoya J., Stefanov I., Vlaeminck B. (2012). Milk odd- and branched-chain fatty acids as biomarkers of rumen function—An update. Anim. Feed Sci. Technol..

[67] Taormina V.M., Unger A.L., Kraft J. (2024). Full-fat dairy products and cardiometabolic health outcomes: Does the dairy-fat matrix matter?. Front. Nutr..

[68] Odongo N.E., Or-Rashid M.M., Kebreab E., France J., McBride B.W. (2007). Effect of supplementing myristic acid in dairy cow rations on ruminal methanogenesis and fatty acid profile in milk. J. Dairy Sci..

[69] Chilliard Y., Martin C., Rouel J., Doreau M. (2009). Milk fatty acids in dairy cows fed whole crude linseed, extruded linseed, or linseed oil, and their relationship with methane output. J. Dairy Sci..

[70] Engelke S.W., Das G., Derno M., Tuchscherer A., Wimmers K., Rychlik M., Kienberger H., Berg W., Kuhla B., Metges C.C. (2019). Methane prediction based on individual or groups of milk fatty acids for dairy cows fed rations with or without linseed. J. Dairy Sci..

[71] Dijkstra J., van Zijderveld S.M., Apajalahti J., Bannink A., Gerrits W.J.J., Newbold J.R., Perdok H.B., Berends H. (2011). Relationships between methane production and milk fatty acid profiles in dairy cattle. Anim. Feed Sci. Technol..

[72] Castro-Montoya J., Campeneere S.D., Baets B.D., Fievez V. (2016). The potential of milk fatty acids as biomarkers for methane emissions in dairy cows: A quantitative multi-study survey of literature data. J. Agric. Sci..

[73] Palmquist D.L., Fox P.F., McSweeney P.L.H. (2006). Milk fat: Origin of fatty acids and influence of nutritional factors thereon. Advanced Dairy Chemistry, Lipids.

[74] Bayat A.R., Tapio I., Vilkki J., Shingfield K.J., Leskinen H. (2018). Plant oil supplements reduce methane emissions and improve milk fatty acid composition in dairy cows fed grass silage-based diets without affecting milk yield. J. Dairy Sci..

[75] Kliem K.E., Morgan R., Humphries D.J., Shingfield K.J., Givens D.I. (2008). Effect of replacing grass silage with maize silage in the diet on bovine milk fatty acid composition. Animal.

[76] Bessa R.J.B., Maia M.R.G., Jerónimo E., Belo A.T., Cabrita A.R.J., Dewhurst R.J., Fonseca A.J.M. (2009). Using microbial fatty acids to improve understanding of the contribution of solid associated bacteria to microbial mass in the rumen. Anim. Feed Sci. Technol..

[77] Abdoul-Aziz S.K.A., Zhang Y., Wang J. (2021). Milk odd and branched chain fatty acids in dairy cows: A review on dietary factors and its consequences on human health. Animals.

[78] de Souza J., Leskinen H., Lock A.L., Shingfield K.J., Huhtanen P. (2020). Between-cow variation in milk fatty acids associated with methane production. PLoS ONE.

[79] Mohammed R., McGinn S.M., Beauchemin K.A. (2011). Prediction of enteric methane output from milk fatty acid concentrations and rumen fermentation parameters in dairy cows fed sunflower, flax, or canola seeds. J. Dairy Sci..

[80] Kaneda T. (1991). Iso- and anteiso-fatty acids in bacteria: Biosynthesis, function, and taxonomic significance. Microbiol. Rev..

[81] Vlaeminck B., Fievez V., Cabrita A.R.J., Fonseca A.J.M., Dewhurst R.J. (2006). Factors affecting odd- and branched-chain fatty acids in milk: A review. Anim. Feed Sci. Technol..

[82] Vlaeminck B., Gervais R., Rahman M.M., Gadeyne F., Gorniak M., Doreau M., Fievez V. (2015). Postruminal synthesis modifies the odd- and branched-chain fatty acid profile from the duodenum to milk. J. Dairy Sci..

[83] van Gastelen S., Antunes-Fernandes E.C., Hettinga K.A., Dijkstra J. (2017). Relationships between methane emission of Holstein Friesian dairy cows and fatty acids, volatile metabolites and non-volatile metabolites in milk. Animal.

[84] Jahreis G., Fritsche J., Steinhart H. (1997). Conjugated linoleic acid in milk fat: High variation depending on production system. Nutr. Res..

[85] Castro-Montoya J.M., Peiren N., Veneman J., De Baets B., De Campeneere S., Fievez V. (2017). Predictions of methane emission levels and categories based on milk fatty acid profiles from dairy cows. Animal.

[86] van Lingen H.J., Crompton L.A., Hendriks W.H., Reynolds C.K., Dijkstra J. (2014). Meta-analysis of relationships between enteric methane yield and milk fatty acid profile in dairy cattle. J. Dairy Sci..

[87] Bougouin A., Appuhamy J.A.D.R.N., Ferlay A., Kebreab E., Martin C., Moate P.J., Benchaar C., Lund P., Eugène M. (2019). Individual milk fatty acids are potential predictors of enteric methane emissions from dairy cows fed a wide range of diets: Approach by meta-analysis. J. Dairy Sci..

[88] Denninger T.M., Schwarm A., Birkinshaw A., Terranova M., Dohme-Meier F., Münger A., Eggerschwiler E., Bapst B., Wegmann S., Clauss M. (2020). Immediate effect of Acacia mearnsii tannins on methane emissions and milk fatty acid profiles of dairy cows. Anim. Feed Sci. Technol..

[89] Dijkstra J., van Gastelen S., Antunes-Fernandes E.C., Warner D., Hatew B., Klop G., Podesta S.C., van Lingen H.J., Hettinga K.A., Bannink A. (2016). Relationships between milk fatty acid profiles and enteric methane production in dairy cattle fed grass- or grass silage-based diets. Anim. Prod. Sci..

[90] Rico D.E., Chouinard P.Y., Hassanat F., Benchaar C., Gervais R. (2016). Prediction of enteric methane emissions from Holstein dairy cows fed various forage sources. Animal.

