# Supplementary material for: Milk Fatty Acids as Potential Biomarkers of Enteric Methane Emissions in Dairy Cattle: A Review
Source: Animals (Basel). 2025 Jul 28;15(15):2212. doi: 10.3390/ani15152212 (PMC12345473; doi:10.3390/ani15152212)
Supplement: Supplementary file 1 [file animals-15-02212-s001.zip › animals-3681036-supplementary.pdf]

Supplementary Table S1. Summary of reported correlations between milk fatty acids (FA) and methane (CH_4) emission metrics and their reported strengths across published studies, including whether the association was positive (+), negative (-), or absent (Ø), and the strength of the correlation (W = weak, M = moderate, S = strong).

	Chilliard et al. [69]		Dijkstra et al. [71]		Mohammed et al.[1] [79]		Engelke et al.[1] [70]		Rico et al. [90]		de Souza et al. [78]	
FA unit:	% total FA		g/100 g total FA		% total FAME		% total lipids		% total FA		g/100 g milk FA	
CH_4 unit:	g/d		g/kg DMI		g/d		L/d		g/d		mmol/mol VFA	
Fatty acid	r	Strength	r	Strength	r	Strength	r	Strength	r	Strength	*β*	*P*-value
**SFA**					+	M	+	S				
4:0	+	S	-	W			+	M	Ø		+	< 0.1
6:0	+	S	+	W			+	S	Ø		Ø	
8:0	+	S	+	M	+	M	+	M	+	W	Ø	
10:0	+	S	+	M			+	M	+	M		
12:0	+	S	+	W			+	W	+	M		
14:0	+	S	-	W			+	M	+	W		
16:0	+	S	+	M			+	M	Ø		Ø	
18:0			Ø				-	W	Ø		Ø	
20:0			Ø				-	W	Ø			
22:0							-	S	Ø			
24:0							+	M	Ø			
**OBCFA**									-	M		
9:0	+	S									-	< 0.01
11:0	+	S	+	M			Ø		Ø			
13:0			-	W			Ø		Ø			
13:0-*iso*									Ø		Ø	
13:0-*anteiso*									-	W	Ø	
14:0-*iso*			+	M			Ø		+	W		
15:0	+	S	+	W	-	W	Ø		-	M	-	< 0.01
15:0-*iso*			+	M			-	M	Ø		Ø	
15:0-*anteiso*			Ø				Ø		Ø		-	< 0.01
16:0-*iso*					+	W	Ø		+	W	Ø	
17:0	+	S	+	W			Ø		-	M	Ø	
17:0-*iso*			-	M			Ø		-	M	Ø	
17:0-*anteiso*			+	M			Ø		Ø		Ø	
17:1 *c*9			-	S	-	S	-	M	-	S		
18:0-*iso*							Ø		Ø		Ø	
**MUFA**							-	S				
10:1	+	S					Ø		Ø			
12:1	+	S					Ø		Ø			
14:1 *c*9			Ø				Ø		-	M		
14:1 *c*11									+	M		
16:1 *t*9							-	W	Ø			
16:1 *t*11	-	S										
16:1 *c*9			Ø				Ø		-	W		
16:1 *c*11									-	M		
16:1 *c*13									+	W		
18:1 *t*4									-	M		
18:1 *t*5									-	M		
18:1 *t*6-*t*8	-	S			-	W			-	W		
18:1 *t*6-*t*9			-	M								

	Chilliard et al. [69]		Dijkstra et al. [71]		Mohammed et al.[1] [79]		Engelke et al.[1] [70]		Rico et al. [90]		de Souza et al. [78]	
FA unit:	% total FA		g/100 g total FA		% total FAME		% total lipids		% total FA		g/100 g milk FA	
CH_4 unit:	g/d		g/kg DMI		g/d		L/d		g/d		mmol/mol VFA	
Fatty acid	r	Strength	r	Strength	r	Strength	r	Strength	r	Strength	*β*	*P*-value
18:1 *t*9			-	M			-	M	-	W		
18:1 *t*10	-	S			-	W			-	M	Ø	
18:1 *t*11	-	S					Ø		Ø		-	0.02
18:1 *t*10+*t*11			-	M								
18:1 *t*12	-	S	-	W					-	W		
18:1 *t*13+*t*14	-	S	+	W					-	M		
18:1 *c*14+*t*16	-	S	-	W	-	W						
18:1 *c*15+*t*17	-	S										
∑ *trans* 18:1	-	S					-	M				
18:1 *c*9	-	S	-	M			-	M	Ø		Ø	
18:1 *c*10	-	S										
18:1 *c*11			+	S	-	S	-	S	-	W	-	< 0.01
18:1 *c*12	-	S	-	M			-	M	Ø			
18:1 *c*13	-	S	+	M	-	M			-	W		
18:1 *c*16					-	M			Ø			
∑ *cis* 18:1	-	S					-	S				
20:1 *c*9									-	M		
20:1 *c*11							Ø		+	W		
**PUFA**					-	M	Ø		-	W		
18:2 *t*8,*c*12									-	M		
18:2 *t*8,*c*13									-	S		
18:2 *c*9,*t*11			-	W			Ø		Ø		-	0.01
18:2 *c*9,*t*12									-	W		
18:2 *c*9,*c*12			-	M	-	M	Ø		Ø			
18:2 *c*9,*t*13	-	S							Ø			
18:2 *t*10,*c*12									-	W		
18:2 *t*11,*c*15	-	S	-	W					-	W		
18:3 *c*6,*c*9,*c*12							-	M	+	W		
18:3 *c*9,*c*12,*c*15			Ø		-	M	-	M	-	M	-	0.01
18:4 *c*6,*c*9,*c*12,*c*15									-	M		
20:2 *c*11,*c*14							Ø		Ø			
20:3 *c*11,*c*14,*c*17									-	M		
20:4 *c*5,*c*8,*c*11,*c*14	+	S					Ø		Ø			
22:3 *c*13,*c*16,*c*19									-	M		
22:5 *c*4,*c*7,*c*10,*c*13,*c*16									-	M		
22:5 *c*7,*c*10,*c*13,*c*16,*c*19							Ø		-	W		
22:6 *c*4, *c*7,*c*10,*c*13,*c*16,*c*19									-	M		

[1] Values derived from the sum of all diets

DMI, dry matter intake; FAME, fatty acid methyl ester; MUFA, monounsaturated fatty acids; OBCFA, odd- and branched-chain fatty acids; PUFA, polyunsaturated fatty acids; SFA, saturated fatty acids; VFA, volatile fatty acids

Supplementary Table S2. Reported correlations between milk fatty acids (FA, g/100 g total FA) and methane (CH_4) emission metrics (Output, g/d; Yield, g/kg dry matter intake; Intensity, g/kg milk unit) across studies. Correlation direction is indicated as positive (+), negative (-), or no correlation (Ø), and strength is categorized as weak (W), moderate (M), or strong (S).

	CH_4 emission	van Lingen et al. [86]		Castro-Montoya et al. [72]		Dijkstra et al. [89]		Castro-Montoya et al. [85]		van Gastelen et al. [83]		Bougouin et al. [87]	
Fatty acid	metric	r	Strength[1]	r	Strength[2]	r	Strength[1]	r	Strength[2]	r	Strength[1]	r	Strength[2]
	Output			+	M-S*								
**SFA**	Yield			Ø									
	Intensity			+	M-S								
	Output			+	M-S					Ø		+	W
4:0	Yield	Ø		Ø		-	W			Ø		+	W
	Intensity	-	W	Ø		-	M			Ø		Ø	
	Output			+	M-S					Ø		+	M
6:0	Yield	+	W	Ø		-	W			Ø		+	W
	Intensity	Ø		+	M-S	Ø				Ø		+	W
	Output			+	M-S					Ø		+	M
8:0	Yield	+	W	+	M-S	Ø				Ø		+	W
	Intensity	Ø		+	M-S	Ø				Ø		+	W
	Output			+	W					Ø		+	M
10:0	Yield	+	W	+	M-S	Ø				Ø		+	W
	Intensity	+	W	+	M-S	+	W			Ø		+	W
	Output									Ø		+	W
12:0	Yield	Ø				Ø				Ø		+	W
	Intensity	+	M			+	M			Ø		+	W
	Output			Ø						Ø		+	M
14:0	Yield	Ø		-	W	+	W			Ø		+	W
	Intensity	+	W	Ø		+	M			Ø		+	W
	Output			+	M-S					Ø		+	M
16:0	Yield	+	M	+	M-S	+	M	+	S	Ø		+	W
	Intensity	+	W	+	M-S	+	S	+	M	Ø		+	W
	Output			+	W					Ø		Ø	
18:0	Yield	Ø		Ø		-	W			Ø		Ø	
	Intensity	-	W	+	W	-	W			Ø		Ø	
	Output			+	M-S					Ø		Ø	
20:0	Yield	Ø		+	M-S	+	W			+	S	Ø	
	Intensity	Ø		+	M-S	+	S			+	M	Ø	
	Output									Ø			
22:0	Yield					+	W			+	M		
	Intensity					+	S			+	S		
	Output									Ø			
24:0	Yield					+	W			+	M		
	Intensity					+	S			+	S		
	Output			+	M-S								
**OBCFA**	Yield			+	M-S								
	Intensity			+	M-S								
	Output							+	W			Ø	
9:0	Yield											-	W
	Intensity											Ø	
11:0	Output			Ø								+	W
	Yield			+	M-S							-	W

Fatty acid	CH_4 emission metric	van Lingen et al. [86] r	van Lingen et al. [86] Strength[1]	Castro-Montoya et al. [72] r	Castro-Montoya et al. [72] Strength[2]	Dijkstra et al. [89] r	Dijkstra et al. [89] Strength[1]	Castro-Montoya et al. [85] r	Castro-Montoya et al. [85] Strength[2]	van Gastelen et al. [83] r	van Gastelen et al. [83] Strength[1]	Bougouin et al. [87] r	Bougouin et al. [87] Strength[2]
	Intensity			+	M-S							Ø	
	Output			+	W							Ø	
13:0	Yield			Ø		+	W					-	W
	Intensity			+	M-S	+	M					Ø	
	Output											+	W
13:0-*iso*	Yield											+	M
	Intensity											+	W
	Output											-	M
13:0-*anteiso*	Yield											+	W
	Output											+	M
	Output			+	M-S					Ø			
14:0-*iso*	Yield	Ø		+	M-S	+	W			-	M		
	Intensity	+	M	+	M-S	+	S			Ø			
	Output			+	M-S					Ø		Ø	
15:0	Yield	Ø		+	M-S	+	M			Ø		Ø	
	Intensity	+	W	+	M-S	+	S			+	M	Ø	
	Output			+	M-S					Ø		Ø	
15:0-*iso*	Yield			+	M-S	+	W			+	M	Ø	
	Intensity			+	M-S	+	S			+	S	Ø	
	Output			+	M-S					Ø		Ø	
15:0-*anteiso*	Yield	Ø		+	M-S	+	W			Ø		+	W
	Intensity	Ø		+	M-S	+	S			Ø		Ø	
	Output			+	M-S					Ø		+	W
16:0-*iso*	Yield	+	W	+	M-S	Ø				Ø		+	W
	Intensity	Ø		+	M-S	+	M			Ø		+	M
	Output			+	M-S					Ø		-	W
17:0	Yield	Ø		+	W	+	M	-	S	+	M	Ø	
	Intensity	Ø		+	M-S	+	M			Ø		+	W
	Output									Ø		-	M
17:0-*iso*	Yield					-	W			Ø		-	W
	Intensity					Ø				Ø		Ø	
	Output			+	M-S					Ø		-	W
17:0-*anteiso*	Yield			+	M-S	Ø				Ø		Ø	
	Intensity			+	M-S	+	W	-	M	Ø		Ø	
	Output							-	S	Ø		-	M
17:1 *c*9	Yield					Ø				Ø		Ø	
	Intensity					-	W			Ø		Ø	
	Output							-	S				
18:0-*iso*	Yield												
	Intensity												
	Output			-	W								
**MUFA**	Yield			Ø									
	Intensity			Ø									
	Output											+	M
10:1	Yield											+	W
	Intensity											+	W
12:1	Output							+	M				

Fatty acid	CH_4 emission metric	van Lingen et al. [86] r	Strength[1]	Castro-Montoya et al. [72] r	Strength[2]	Dijkstra et al. [89] r	Strength[1]	Castro-Montoya et al. [85] r	Strength[2]	van Gastelen et al. [83] r	Strength[1]	Bougouin et al. [87] r	Strength[2]
	Yield												
	Intensity												
14:1 *c*9	Output			Ø						+	S	Ø	
	Yield	Ø		-	W	+	W			Ø		Ø	
	Intensity	+	W	-	M-S	+	M			+	M	Ø	
16:1 *t*9	Output									Ø			
	Yield									Ø			
	Intensity									-	M		
16:1 *t*11	Output			Ø									
	Yield			Ø									
	Intensity			Ø									
16:1 *t*14	Output												
	Yield												
	Intensity							+	M				
16:1 *c*9	Output									Ø		-	W
	Yield					Ø		+	M	Ø		-	W
	Intensity					-	W	+	M	Ø		-	W
16:1 *c*11	Output			Ø									
	Yield			+	W								
	Intensity			+	M-S								
18:1 *t*6-*t*8	Output									-[3]	M	-	M
	Yield	-[4]	W			-	M			Ø		-	M
	Intensity	Ø[4]				Ø				Ø		-	M
18:1 *t*9	Output									-	M	-	W
	Yield					-	M			-	S	-	W
	Intensity					-	M			Ø		-	W
18:1 *t*10	Output									-	M	-	M
	Yield					-	M			-	S	-	M
	Intensity					Ø				-	M	-	M
18:1 *t*11	Output									-	S	-	W
	Yield					-	W			-	S	-	W
	Intensity					Ø				Ø		-	W
18:1 *t*10+*t*11	Output											-	M
	Yield	-	S									-	M
	Intensity	-	W									-	M
18:1 *t*12	Output			-	M-S							-	W
	Yield			Ø								-	W
	Intensity			-	M-S							-	W
18:1 *t*13+*t*14	Output			-	M-S			-[5]	S			-	M
	Yield			-	M-S			-[5]	S			-	W
	Intensity			-	M-S							-	W
18:1 *c*11+*t*15	Output							-[6]	M	-	M	-[6]	M
	Yield					-	M			-	M	-[6]	M
	Intensity					-	S			-	M	-[6]	M
∑ *trans* 18:1	Output												
	Yield	-	S										
	Intensity	-	W										

Fatty acid	CH4 emission metric	van Lingen et al. [86] r	Strength[1]	Castro-Montoya et al. [72] r	Strength[2]	Dijkstra et al. [89] r	Strength[1]	Castro-Montoya et al. [85] r	Strength[2]	van Gastelen et al. [83] r	Strength[1]	Bougouin et al. [87] r	Strength[2]
	Output									Ø		-	W
18:1 *c*9	Yield	Ø				-	W			Ø		-	W
	Intensity	-	M			-	S			Ø		Ø	
	Output											-	S
18:1 *c*10	Yield											-	M
	Intensity											-	M
	Output			Ø								-	M
18:1 *c*11	Yield	-	S	Ø								-	M
	Intensity	-	M	-	W							-	W
	Output			Ø						-	M	Ø	
18:1 *c*12	Yield	-	M	Ø		-	S			-	S	-	W
	Intensity	Ø		Ø		-	M			-	M	-	W
	Output			-	M-S					Ø			
18:1 *c*13	Yield	-	W	-	M-S	-	W			Ø			
	Intensity	Ø		-	M-S	-	M	-	M	Ø			
	Output											+	M
18:1 *c*14	Yield											Ø	
	Intensity											Ø	
	Output											-	M
18:1 *c*15	Yield							+	M			-	M
	Intensity											-	W
	Output												
∑ *cis* 18:1	Yield	-	S										
	Intensity	-	M										
	Output											-	W
20:1 *c*9	Yield											Ø	
	Intensity											Ø	
	Output									Ø			
20:1 *c*11	Yield					-	W			Ø			
	Intensity					-	S			Ø			
	Output			Ø									
**PUFA**	Yield			Ø									
	Intensity			-	W								
	Output									-	S	-	W
18:2 *c*9,*t*11	Yield					-	W			-	S	-	W
	Intensity					Ø				Ø		-	W
	Output									-	S	-	M
18:2 *c*9,*c*12	Yield	-	W			-	M			-	S	-	W
	Intensity	-	W			-	W			Ø		-	W
	Output											-	W
18:2 *c*9,*t*13	Yield											-	W
	Intensity											-	W
	Output											-	W
18:2 *t*11,*c*15	Yield											-	W
	Intensity											-	W
18:3 *c*6,*c*9,*c*12	Output									-	S	+	W
	Yield					-	W	-[7]	S	-	S	+	W

Fatty acid	CH4 emission metric	van Lingen et al. [86] r	Strength[1]	Castro-Montoya et al. [72] r	Strength[2]	Dijkstra et al. [89] r	Strength[1]	Castro-Montoya et al. [85] r	Strength[2]	van Gastelen et al. [83] r	Strength[1]	Bougouin et al. [87] r	Strength[2]
	Intensity					Ø		-[7]	M	Ø		Ø	
	Output									Ø			
18:3 *c*9,*c*12,*c*15	Yield	Ø				-	S			+	M		
	Intensity	Ø				-	W			Ø			
	Output									Ø			
20:2 *c*11,*c*14	Yield					+	W			Ø			
	Intensity					+	S			Ø			
	Output									-	M		
20:3 *c*8,*c*11,*c*14	Yield					Ø				Ø			
	Intensity					+	M			Ø			
	Output									-	M	Ø	
20:4 *c*5,*c*8,*c*11,*c*14	Yield					Ø				-	S	Ø	
	Intensity					+	S			Ø		+	W
	Output									+	M		
20:4 *c*8,*c*11,*c*14,*c*17	Yield					-	W			Ø			
	Intensity					Ø				Ø			
	Output									Ø			
20:5 *c*5,*c*8,*c*11,*c*14,*c*17	Yield					-	W			Ø			
	Intensity					+	W			+	M		
	Output									Ø			
22:5 *c*7,*c*10,*c*13,*c*16,*c*19	Yield					-	W			Ø			
	Intensity					+	S			+	M		

[1] CH_4 intensity: g/kg fat-and-protein corrected milk

[2] CH_4 intensity: g/kg milk

[3] Only 18:1 *t*6

[4] Includes 18:1 *t*6-*t*9

[5] Only 18:1 *t*13

[6] Only 18:1 *t*15

[7] Includes 18:3 *c*6,*c*9,*c*12 + 20:0

* M-S indicates moderate to strong correlation

MUFA, monounsaturated fatty acids; OBCFA, odd- and branched-chain fatty acids; PUFA, polyunsaturated fatty acids; SFA, saturated fatty acids; VFA, volatile fatty acids
